# A literature review of major surgery experience with emicizumab in people with hemophilia A without factor VIII inhibitors

**DOI:** 10.1016/j.rpth.2025.102693

**Published:** 2025-01-31

**Authors:** Giancarlo Castaman, Stacy E. Croteau, Doris Quon, Lucy Lee, Letizia Polito, Víctor Jiménez-Yuste

**Affiliations:** 1 Department of Oncology, Centre for Bleeding Disorders and Coagulation, Careggi University Hospital, Florence, Italy; 2 Boston Children’s Hospital, Boston Hemophilia Center, Boston, Massachusetts, USA; 3 Orthopedic Hemophilia Treatment Center at Orthopedic Institute for Children Los Angeles, Los Angeles, California, USA; 4 Genentech, Inc., South San Francisco, California, USA; 5 F. Hoffmann-La Roche Ltd, Basel, Switzerland; 6 Hematology Department, Hospital Universitario La Paz-IdiPaz, Autónoma University, Madrid, Spain

**Keywords:** emicizumab, general surgery, hemophilia A, hemorrhage, review, safety

## Abstract

People with hemophilia A have a total or partial deficiency of factor (F)VIII, causing spontaneous and/or traumatic bleeding into the joints, muscles, and soft tissues. Major surgery may be required to restore joint mobility or treat the symptoms of common comorbidities in people with hemophilia A. Additional factor replacement is recommended during the perioperative period; collated information on the experience of emicizumab-treated people with hemophilia A during major surgery is currently lacking. To provide a consolidated narrative summary of the experience with emicizumab in people with hemophilia A without FVIII inhibitors undergoing major surgery, a comprehensive literature search was performed using PubMed/MEDLINE (cut-off date: March 31, 2024); the abstract books for applicable congresses (2016–2024) were searched manually. Studies were included if reporting original data on people with hemophilia A of all ages and hemophilia A severities without FVIII inhibitors on emicizumab prophylaxis who had undergone major surgery. Outcomes collected included perioperative surgical management, adverse events, and bleeding events. Twenty publications were included; 72 procedures were reported. Twenty-two orthopedic and 34 other major procedures were specifically described. FVIII replacement was used to manage 66 procedures perioperatively, and 25 procedures were managed in conjunction with antifibrinolytics. Fifteen procedures resulted in a bleeding event, and one individual experienced a thrombotic event. No deaths were reported. This review provides a consolidated narrative of the currently reported experiences of emicizumab-treated people with hemophilia A without FVIII inhibitors undergoing major surgery, helping to support the future management decisions of emicizumab-treated people with hemophilia A during surgery.

## Essentials

• Data on emicizumab-treated people with hemophilia A during major surgery are limited.
• Perioperative management, bleeding outcomes, and AEs in the literature were explored.
• Of the 72 procedures reported, 66 were managed with FVIII replacement perioperatively.
• Across the 20 included publications, 15 procedures resulted in bleeding events and 1 thrombotic event occurred.

## Visual Abstract

**Figure:**
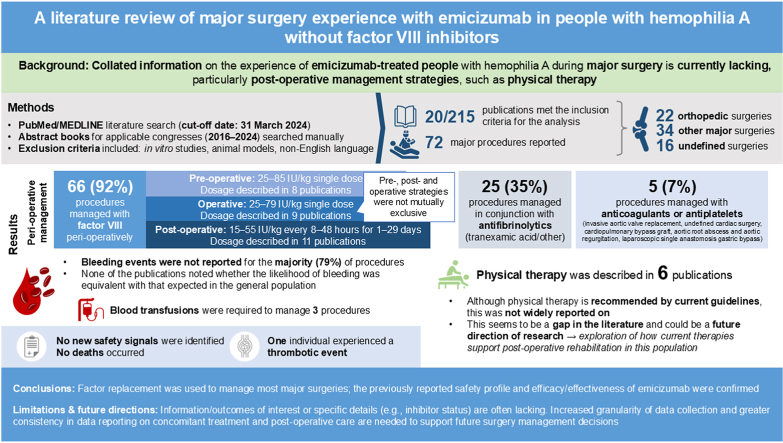

## 1 Introduction

Total or partial factor (F)VIII deficiency in people with congenital hemophilia A can result in spontaneous and/or traumatic bleeding into the joints, muscles, and soft tissues [1]. Hemophilia A severity is classified as mild, moderate, or severe, based on the endogenous plasma FVIII concentrations of >5 to 40 IU/dL, 1 to 5 IU/dL, or <1 IU/dL, respectively [1,2].

Hemophilic arthropathy and/or chronic synovitis is caused by repetitive bleeding events into the joints and surrounding tissues, and is common in people with severe hemophilia A or with a severe bleeding phenotype [3]. The debilitating effects of these conditions include pain and reduced mobility, which negatively impact quality of life and can lead to disability [3,4].

People with hemophilia A could require major surgery, and often orthopedic intervention may be required for some people with hemophilia A to restore joint mobility, reduce pain, and in some cases, prevent the development of, and/or treat, degenerative joint disease [5]. In addition, other major surgical procedures not related to hemophilia A, or due to specific comorbidities, such as cardiovascular disease, may be required [2]. Typically, people with hemophilia A who are treated on demand or prophylactically with FVIII will require additional factor replacement if undergoing major surgery to prevent excessive and persistent bleeding before, during, and after the procedure [2].

Emicizumab, a recombinant, humanized, bispecific monoclonal antibody, is administered subcutaneously, and mimics the cofactor function of activated FVIII by bridging activated FIX and FX [6]. Due to its half-life of 4 to 5 weeks [7], emicizumab requires less frequent dosing than factor replacements [6,8], and has the ability to improve hemostasis regardless of the presence of FVIII inhibitors as it does not share any sequence homology with FVIII [6]. Emicizumab is approved for routine prophylaxis to prevent or reduce the frequency of bleeding episodes in people with hemophilia A of all ages, both with and without FVIII inhibitors [8]. The safety and efficacy of emicizumab has been demonstrated in adult and pediatric hemophilia A populations with and without FVIII inhibitors in the HAVEN clinical trial program [[9], [10], [11], [12], [13], [14]] and in real-world studies [[15], [16], [17], [18], [19], [20]]. Perioperative management for people with hemophilia A who are currently treated with emicizumab may differ due to its sustained therapeutic plasma levels [2,9].

Although Jiménez-Yuste et al. [21] previously presented and described the experiences of people with hemophilia A with FVIII inhibitors treated with emicizumab during surgery in the current literature, collated information regarding surgeries for people with hemophilia A without FVIII inhibitors using emicizumab does not currently exist. The objective of this article is to provide a consolidated source of the experience with emicizumab in people with hemophilia A without FVIII inhibitors undergoing major surgical procedures to date. This review will focus on the types of major surgeries performed and perioperative (ie, the days and weeks before, during, and after surgery) use of factor replacement. Other key outcomes assessed will include the use of antifibrinolytics and/or anticoagulants, bleeding events and blood loss, and thrombotic events and death. The use of physical therapy following surgery will also be explored.

## 2 Methods

### 2.1 Literature search

A comprehensive literature search was performed using PubMed/MEDLINE from inception to the cut-off date of March 31, 2024. The abstract books for applicable congresses, between the years 2016 and 2024, that have a focus on blood disorders, were searched manually. These comprised: the American Society of Hematology, European Association for Haemophilia and Allied Disorders, International Society on Thrombosis and Haemostasis and World Federation of Hemophilia (WFH). A combination of indexing and free text terms for hemophilia A, emicizumab, prophylaxis, and surgery were used for the search strategy (Supplementary Table S1). Studies were included if reporting original primary data on people with hemophilia A without inhibitors against FVIII on emicizumab prophylaxis who had undergone major surgery. All ages and disease severities were included. Major surgery was defined as per the definition by Jiménez-Yuste et al. 2021 [21]: “arthroplasty (including joint replacement, fusion, and endoprosthesis), open reduction and internal fixation, abdominal surgery, thoracic surgery, neurologic surgery, “–ectomy” procedures, and “–otomy” procedures.”

Any type of study was included (clinical trials, registries, and other types of real-world data studies, such as observational studies, claims database reports, and e-health records). To fulfill the inclusion criteria, publications must have been in English language. Studies that reported on *in vitro* models, animal models, cell lines, and other molecules were excluded. Publications that focused on an inherited or acquired bleeding disorder other than congenital hemophilia A were also excluded. Publications that reported on both people with hemophilia A with and without FVIII inhibitors were excluded if the outcomes of interest reported did not distinguish between those with or without FVIII inhibitors. Publications that presented a trial in progress, and therefore did not report on outcomes, were also excluded. In the instance of duplication of data, the latest publication for each study/data set was included unless significantly more data were available in the earlier publication.

To be included, publications must have reported at least one, but not necessarily all, of the following outcomes: preoperative, operative, or postoperative surgical management (including FVIII activity levels at baseline, type, dosage and administration frequency of factor replacement, total factor consumption, use of antifibrinolytics [tranexamic acid and/or aminocaproic acid], and anticoagulant/antiplatelet use [including type, dosage, administration frequency]), type and duration of physical therapy, duration of hospitalization, adverse events (AEs; including, but not limited to, adverse events of special interest [AESIs], such as thrombotic events and thrombotic microangiopathy), number of blood transfusions, number of bleeding events (including blood loss), or death.

### 2.2 Screening and data extraction

The screening and data extraction processes were performed manually. All texts underwent independent screening by 2 reviewers according to the eligibility criteria defined in the protocol, and any disagreements were resolved by a third reviewer. All data were manually extracted by one individual and a consensus check was performed by a different individual. Patient demographics collected included age, weight at time of surgery, hemophilia A severity, comorbidities, immune tolerance induction history, prescribed emicizumab dose and administration frequency, and date of last emicizumab dose before surgery. Type of major surgery, general anesthesia, and requirement for respiratory assistance were also collected, where possible. Outcomes of interest included perioperative surgical management (factor replacement and/or antifibrinolytics and/or anticoagulants), physical therapy, bleeding, blood loss, and AEs, including AESIs. All preselected outcomes searched for during data extraction are presented in Table 1.

**Table 1:** Preselected outcomes prior to data extraction.

Preselected outcomes∗
**Patient demographics at baseline**
Age
Weight at time of surgery
Hemophilia A severity
Comorbidities/history of immune tolerance induction therapy
Prescribed emicizumab dose and administration frequency
Date of last emicizumab dose before surgery
**Surgery details**
Type of major surgery
Setting
General anesthesia and/or respiratory assistance
**Perioperative management (before, during, and after surgery)**
Factor VIII levels
Factor replacement
Type
Dosage
Administration frequency
Cumulative consumption
Use of antifibrinolytics and/or anticoagulants and/or antiplatelets
Type
Dosage
Administration frequency
Bleeding events
Adverse events
Thrombotic events and thrombotic microangiopathies
Death
**Additional outcomes of interest**
Type and duration of physical therapy
Hospitalization time
Number of blood transfusions
Blood loss
Mechanical intervention for the prevention of thrombotic events

∗ Not all outcomes were reported in the included publications.

### 2.3 Analysis

A narrative synthesis was used to explain the findings. Due to the broadness and variety of the literature search results, no meta-analysis could be performed.

## 3 Literature Search Results

### 3.1 Descriptive characteristics

Overall, 215 publications were identified through database and manual searches. Following screening, 20 publications fulfilled the inclusion criteria and were included in the overall review (Figure). One publication [22] reported on a subset of people with hemophilia A already described in a later publication [23], but in greater detail, so in order to avoid losing the granularity provided for this subset, it was included in the narrative part of the review only. Nineteen publications reporting on primary data were therefore included in the analysis. One (5.3%) of these reported on clinical trial data [23], and 18 (94.7%) reported data from the real-world setting [[24], [25], [26], [27], [28], [29], [30], [31], [32], [33], [34], [35], [36], [37], [38], [39], [40], [41]] (Table 2). Two (10.5%) publications focused on pediatric and adolescent people with hemophilia A (<18 years) [27,28], 8 (42.1%) focused on adults with hemophilia A (≥18 years) [24,25,[30], [31], [32], [33],^35^,^41^], and 5 (26.3%) focused on all age groups [23,26,[36], [37], [38]]. Four (21.1%) publications did not state the ages of all people with hemophilia A involved in the analysis [29,34,39,40].

**Figure:**
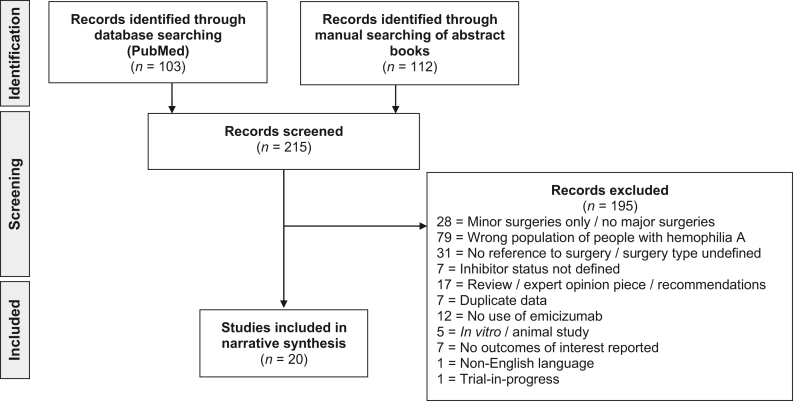
Search results: number of records identified, screened and included in the review.

**Table 2:** Reported major surgeries in people with hemophilia A without factor VIII inhibitors receiving emicizumab.

Publication	Patient details	Surgery type	Perioperative management	Complications and management strategy	Outcome of management strategy
Abraham et al. 2023 [39]	18 procedures in total (including the people with hemophilia A below)	Major surgery	**Preoperative:** 35-40 IU/kg SHL rFVIII 2–3 h before surgery. **Operative:** 15-20 IU/kg SHL rFVIII once, if required. **Postoperative:** 15-20 IU/kg SHL rFVIII every 12 h from day 1 to day 5.	Eight breakthrough bleeding events	No thromboembolism or thrombotic microangiopathies occurred
	Person with severe hemophilia A	Right total knee replacement	**Preoperative:** 35-40 IU/kg SHL rFVIII 2–3 h before surgery. **Postoperative:** 15-20 IU/kg SHL rFVIII every 12 h from day 1 to day 5. One additional dose of SHL rFVIII on day 2.	One breakthrough bleed	No thromboembolism or thrombotic microangiopathies occurred
	Person with severe hemophilia A	B/L total knee replacement	**Preoperative:** 35-40 IU/kg SHL rFVIII 2–3 h before surgery. **Postoperative:** 15-20 IU/kg SHL rFVIII every 12 h from day 1 to day 5. Two additional doses of SHL rFVIII on day 12 and day 16.	Two breakthrough bleeds were treated with SHL rFVIII	No thromboembolism or thrombotic microangiopathies occurred
	Person with severe hemophilia A	Left total knee replacement	**Preoperative:** 35-40 IU/kg SHL rFVIII 2–3 h before surgery. **Postoperative:** 15-20 IU/kg SHL rFVIII every 12 h from day 1 to day 5. Two additional doses of SHL rFVIII on day 16 and day 17.	One breakthrough bleed	No thromboembolism or thrombotic microangiopathies occurred
	Person with severe hemophilia A	Right thigh pseudotumor above-knee amputation	**Preoperative:** 35-40 IU/kg SHL rFVIII 2-3 h before surgery. **Postoperative:** 15–20 IU/kg SHL rFVIII every 12 h from day 1 to day 5. Additional SHL rFVIII twice a day from day 11 to day 14.	One stump bleed	No thromboembolism or thrombotic microangiopathies occurred
	Person with severe hemophilia A	B/L total knee replacement	**Preoperative:** 35-40 IU/kg SHL rFVIII 2-3 h before surgery. **Postoperative:** 15-20 IU/kg SHL rFVIII every 12 h from day 1 to day 5. One additional dose of SHL rFVIII on day 9, twice-daily SHL rFVIII from day 13 to day 15, and 1 dose on day 29.	Three breakthrough bleeds	No thromboembolism or thrombotic microangiopathies occurred
Brakta et al. 2023 [30]	Four people with severe HA	Major surgery	**Preoperative:** rFVIII **Postoperative:** rFVIII	Not stated	Not stated
Buckner et al. 2023 [38]	Eight procedures, mean age at first surgery 22.5 y (SD: 18.9). Hemophilia A severity not defined for people with hemophilia A without FVIII inhibitors undergoing major surgery	Major surgery (including total knee joint replacement, reconstruction of anterior cruciate ligament of the knee)	**Postoperative:** Two surgeries were managed with additional factor replacement and were associated with a treated bleed.	Two bleeding events reported, and were treated with SHL rFVIII concentrate	Not stated
Castaman et al. 2023 [37]	28 y of age with severe hemophilia A	Total knee replacement with forced extension on postoperative day 9	**Preoperative:** 70 IU/kg SHL rFVIII. **Postoperative:** Factor replacement (administered to achieve 50 IU/dL for 7-14 days postsurgery): 43 IU/kg every 12 h for 3 d; 43 IU/kg every 24 h for 5 d; 43 IU/kg every 12 h for 1 d; 28 IU/kg every 12 h for 3 d; 14 IU/kg every 12 h for 2 d; 14 IU/kg every 24 h for 7 d. Adjunctive i.v. TXA: 1 g every 12 h for 7 d.	Rehabilitation without FVIII coverage	Hospitalization days were consistent with the type of surgery. No bleeding episodes (bleeding was as expected) and no thromboembolic events occurred.
	63 y of age with severe hemophilia A	*Surgery 1:* Pseudotumor of thigh biopsy *Surgery 2:* Pseudotumor of thigh excision	**Preoperative:** *Surgery 1:* 66 IU/kg SHL rFVIII *Surgery 2:* 85 IU/kg SHL rFVIII **Postoperative:** Factor replacement (administered to achieve 50 IU/dL for 7-14 days postsurgery): *Surgery 1:* 50 IU/kg every 24 h for 3 d; 50 IU/kg every 48 h for 4 d *Surgery 2:* 50 IU/kg every 12 h for 1 d; 30 IU/kg every 12 h for 6 d; 50 IU/kg every 24 h for 7 d. *Both surgeries:* Adjunctive i.v. TXA: 1 g every 12 h for 7 d.	Not stated	
	47 y of age with severe hemophilia A	Splenectomy	**Preoperative:** 70 IU/kg SHL rFVIII. **Postoperative:** Factor replacement (administered to achieve 50 IU/dL for 7-14 d postsurgery): 35 IU/kg every 12 h for 2 d; 23 IU/kg every 12 h for 4 d; 35 IU/kg every 24 h for 7 d. Adjunctive i.v. TXA: 1 g every 8 h for 7 d.		
	5 y of age with severe hemophilia A	Cleft palate correction (second operation)	**Preoperative:** 55 IU/kg SHL rFVIII. **Postoperative:** Factor replacement (administered to achieve 50 IU/dL for 7-14 days postsurgery): 55 IU/kg every 12 h for 1 d; 55 IU/kg every 24 h for 6 d; 55 IU/kg every 48 h for 7 d. Oral TXA: 0.5 g every 8 h for 14 d.		
	39 y of age with severe hemophilia A	Turbinate reduction	**Preoperative:** 25 IU/kg EHL rFVIII. **Postoperative:** Factor replacement (administered to achieve 50 IU/dL for 7-14 days postsurgery): 25 IU/kg every 24 h for 2 d. Oral TXA: 1 g every 8 h for 7 d.		
Cohen et al. 2023 [40]	Eight people with hemophilia A in total (including 3 people with hemophilia A defined below)	Major surgery	**Perioperative:** Antifibrinolytics used for all individuals (excluding those undergoing urological procedures). **Operative:** 50 IU/kg FVIII. **Postoperative:** 50 IU/kg FVIII.	Not specifically stated for major surgeries	No thrombotic complications
	27 y of age with NASH and severe HA	Laparoscopic single anastomosis gastric bypass	**Perioperative:** Unspecific antifibrinolytic. **Operative:** 50 IU/kg rFVIII. **Postoperative:** 50 IU/kg rFVIII; single dose of low molecular weight heparin.	Major bleeding of gastrointestinal origin; heparin treatment was stopped due to bleeding.	No thrombotic complications or deaths
	37 y of age	Retrograde intrarenal surgery with stent insertion	**Operative:** 50 IU/kg rFVIII. **Postoperative:** 50 IU/kg rFVIII.	Clinically relevant non-major bleeding Hematuria; additional FVIII doses were avoided.	No thrombotic complications or deaths
	36 y of age	Total hip replacement	**Perioperative:** Unspecific antifibrinolytic. **Operative:** 50 IU/kg rFVIII. **Postoperative:** 50 IU/kg rFVIII.	Major bleed 1 wk after surgery; daily administration of FVIII for 11 d at home postsurgery.	No thrombotic complications or deaths
Rener et al. 2023 [41]	56 y of age with severe hemophilia A and long-lasting chronic osteomyelitis. Multiple surgical and antibiotic treatment attempts failed. A hyperbaric chamber treatment also failed	Surgery 1: Necrectomy of chronic osteomyelitis with re-arthrodesis (elective) Surgery 2: Below-knee amputation (elective)	**Preoperative:** Surgery 1: 40 IU/kg rFVIII Surgery 2: 37 IU/kg rFVIII Both surgeries: 1 g i.v. TXA on the day of the procedure **Postoperative:** Both surgeries: rFVIII (to maintain a trough level of 0.80-1.00 IU/mL for 3-5 days).	Not stated	No bleeding events occurred Leg prosthesis
	56 y of age with severe hemophilia A	Surgery 1: Bilateral osteosynthesis of the tibia and fibula (urgent) Surgery 2: Below-knee amputation (required due to inflammation persistence)	**Preoperative:** Surgery 1: 53 IU/kg rFVIII Surgery 2: 26 IU/kg rFVIII Both surgeries: 1 g i.v. TXA on the day of the procedure **Operative:** 79 IU/kg EHL rFVIII **Postoperative:** Surgery 1: Mean 65 IU/kg EHL rFVIII per day for 3 d (to maintain a trough level of 0.80-1.00 IU/mL for 3-5 d)	A severe bleeding event was treated with a transfusion of blood products, including red blood cells, fresh frozen plasma and platelets	Remained disabled
	51 y of age with severe hemophilia A	Surgery 1: External ventricular drainage after intraventricular hemorrhage (urgent) Surgery 2: Osteosynthesis after hip fracture (urgent)	**Preoperative:** Surgery 1: 53 IU/kg rFVIII Surgery 2: 27 IU/kg rFVIII Both surgeries: 1 g i.v. TXA on the day of the procedure **Postoperative:** Both surgeries: rFVIII (to maintain a trough level of 0.80-1.00 IU/mL for 3-5 d) Surgery 2: 21 IU/kg rFVIII every other day	Balance instability and persistent cognitive impairment after Surgery 1; intensive physical therapy was required; emicizumab was discontinued	Switched to FVIII prophylaxis Improved physically and cognitively
	52 y of age with severe hemophilia A	Total right knee arthroplasty (elective)	**Preoperative:** 50 IU/kg rFVIII; 1 g i.v. TXA on the day of the procedure. **Postoperative:** rFVIII (to maintain a trough level of 0.80-1.00 IU/mL for 3-5 d).	Sepsis	No hemorrhagic complications Mobility improved, and less pain
Sreedhar et al. 2023 [31]	Early 50s with severe hemophilia A receiving 105 mg/wk emicizumab. Diagnosed with severe mitral regurgitation following hospitalization with atrial fibrillation and cardiac failure	Cardiac surgery	**Preoperative:** 85 IU/kg SHL FVIII. **Operative:** 30 IU/kg SHL FVIII while on bypass circuit; additional heparin boluses were required during surgery **Postoperative:** A continuous infusion of SHL FVIII used to maintain FVIII:C >100 IU/dL.	High blood output was noted 6 h postsurgery; intervention was not required A left atrial appendage thrombus was diagnosed on day 14; enoxaparin followed by edoxaban and a SHL FVIII bolus were administered twice a day	Not stated
Tamijarassane et al. 2023^a^ [29]	Severe hemophilia A with multiple comorbidities, including extensive joint arthropathy, high blood pressure, and moderate aortic stenosis	Invasive aortic valve replacement	**Preoperative:** 3000 IU rFVIII on the morning of the surgery **Operative:** 3000 IU rFVIII; continuous 3 IU/kg/h infusion. **Postoperative:** 3000 IU rFVIII; continuous 3 IU/kg/h infusion until post-op day 7; antiplatelet immediately following the procedure.	Not stated	Patient discharged on bolus dose of FVIII
Carulli et al. 2022 [34]	Two people with severe hemophilia A	Not stated	**Preoperative:** rFVIII **Postoperative:** rFVIII	Not stated	Effective bleeding control. Discharged after early rehabilitation and sent to the rehabilitative ward.
Cusano et al. 2022 [33]	54 y of age with severe hemophilia A receiving 105 mg emicizumab (off-label). Comorbidities included Child–Pugh B cirrhosis, hepatitis C (treated), HIV on antiretroviral therapy (with undetectable viral load), remote portal vein thrombosis, gastrointestinal bleed (treated) and diagnosis of Group B Streptococcus infective endocarditis	Aortic root abscess and aortic regurgitation requiring intervention	**Preoperative:** 60 IU/kg moroctocog alfa to achieve ∼120% FVIII level. **Operative:** Heparin (4 doses): 28,000 U × 1, 10,000 U × 3; 25 IU/kg moroctocog alfa following a generalized sternal ooze; administered once 4.75 h after the start of the surgery; TXA. **Postoperative:** Moroctocog alfa for 14 d: day 1: 30 IU/kg every 12 h + 25 IU/kg × 1 day 2, 3: 40 IU/kg every 12 h day 6, 7: 30 IU/kg every 12 h day 10, 14: 30 IU/kg every 24 h	Persistent oozing from the sternum occurred; hemostasis was achieved with cautery and sutures (post-op chest tube blood loss of 1600 mL)	No further bleeding or thrombotic events occurred.
Dos Santos Ortas et al. 2022 [32]	55 y of age with severe hemophilia A receiving 1.5 mg/kg once weekly emicizumab	Ankle arthrodesis with osteosynthesis due to severe hemophilic arthropathy	**Preoperative:** 40 IU/kg pdFVIII/VWF. **Operative:** 1 × 40 IU/kg pdFVIII/VWF. **Postoperative:** pdFVIII/VWF.	Not stated	Discharged on post-op day 4 due to the absence of bleeding events. No bleeding events or thrombotic events
	52 y of age with severe hemophilia A receiving 6 mg/kg once every 4 wk emicizumab. Previous HIV infection and lymphoma in complete remission	Cubital tunnel syndrome secondary to hemophilic arthropathy that required surgery	**Preoperative:** 50 IU/kg EHL rFVIII × 1. **Operative:** 50 IU/kg EHL rFVIII × 1. **Postoperative:** EHL rFVIII × 1 on post-op day 3.	Not stated	Discharged on post-op day 2 due to the absence of bleeding events. No bleeding events or thrombotic events
Kruse-Jarres et al. 2022 [23]	Eight people with hemophilia A receiving 1.5 mg/kg once weekly, 3 mg/kg once every 2 wk or 6 mg/kg once every 4 wk emicizumab	Major surgery	**Perioperative:** All 8 procedures were managed with additional factor concentrate.	Not stated	No treated bleeds No thrombotic events, thrombotic microangiopathies, deaths, or new FVIII inhibitor development were reported.
Rener et al. 2022 [35]	Two people with hemophilia A (FVIII inhibitor status not defined)	Major surgery	SHL rFVIII for people with hemophilia A without FVIII inhibitors.	Not stated	No thromboembolic events
Guillaume et al. 2021 [24]	44 y of age with severe hemophilia A, receiving emicizumab at a dose of 6 mg/kg once every 4 wk. Previous chronic hepatitis C virus infection (cured in 2017). Previous synoviorthesis of the right elbow and total joint replacement of the right knee under continuous infusion with SHL FVIII	Total arthroplasty of the elbow	**Preoperative:** 4000 IU rFVIII. **Operative:** Continuous infusion of 4 IU/kg/h (280 IU/h) rFVIII, maintained for 24 h. **Postoperative:** 225 IU/h rFVIII infusion for 4 d.	Not stated	No bleeding complications occurred
Hassan & Motwani 2021 [28]	8.6 y of age	Cleft palate correction	**Preoperative:** 25 IU/kg rFVIII; TXA. **Postoperative:** 25 IU/kg rFVIII twice daily for 1 d, then once daily for 4 d; TXA.	Not stated	No bleeding events or thrombotic complications
Isaacs et al. 2021 [25]	57 y of age with severe hemophilia A. Previously diagnosed with HIV, hepatitis C (treated), follicular lymphoma (treated), non-ST-elevation myocardial infarction and high-grade CAD. >5 prior major orthopedic surgeries. Treated with 1.5 mg/kg emicizumab 5 d prior to surgery	Cardiopulmonary bypass grafting surgery	**Preoperative:** 81 mg aspirin. Treatment with emicizumab alone; rFVIII available on standby **Operative:** 35,000 U (400 U/kg) heparin 55 mins into surgery, 275 mg protamine 3 h 50 mins into surgery, and 50 IU/kg rFVIII 4 h 3 mins into surgery. **Postoperative:** Continuous 2 IU/kg/h infusion of rFVIII for 5 d, followed by twice-daily bolus dosing for 5 d 81 mg daily aspirin.	The estimated blood loss was 300 mL	No hemorrhagic complications were documented. The individual was discharged.
Lewandowska et al. 2021 [26]	>12-18 y of age with mild hemophilia A receiving 3 mg/kg once every 2 wk emicizumab	Right medial patellofemoral ligament reconstruction	**Preoperative:** 1 × 50 IU/kg EHL rFVIII. **Postoperative:** EHL rFVIII: 40 IU/kg every 8 h × 3; 40 IU/kg every 12 h × 2; 40 IU/kg daily through post-op day 5 (the final dose was not administered due to higher than expected FVIII trough levels).	Not stated	No bleeds, thrombotic events, thrombotic microangiopathies, or death
	>12-18 y with severe hemophilia A receiving 1.5 mg/kg once weekly emicizumab	Open reduction and internal fixation 5th phalangeal	**Preoperative:** 1 × 50 IU/kg EHL rFVIII. **Postoperative:** 2 × 50 IU/kg EHL rFVIII every 12 h.		No bleeds, thrombotic events, thrombotic microangiopathies, or death
	>18-65 y with moderate hemophilia A receiving 6 mg/kg once every 4 wk emicizumab	Elbow arthroscopic synovectomy with nerve transposition	**Preoperative:** 1 × 50 IU/kg rFVIII. **Postoperative:** rFVIII once daily.	One bleed occurred postoperatively into the soft tissue/muscles of the left forearm on post-op day 8. Additional 50 IU/kg factor replacement × 5 was used to treat this bleed; 6 additional daily doses of 40 IU/kg rFVIII were used on post-op days 8-13.	No major bleeds, thrombotic events, thrombotic microangiopathies, or death
	>18-65 y with severe hemophilia A receiving 6 mg/kg once every 4 wk emicizumab	Screw fixation for a femoral neck fracture	**Preoperative:** 1 × 50 IU/kg EHL rFVIII. **Postoperative:** 32 IU/kg EHL rFVIII on post-op Days 1–7; 38 IU/kg EHL rFVIII on post-op day 9.	Not stated	No bleeds, thrombotic events, thrombotic microangiopathies, or death
McCary et al. 2020 [27]	0.16 y with severe hemophilia A	Intracranial ventricular shunt revision	**Perioperative:** 73 μg/kg pd VWF × 12; multiple doses of FVIII (to maintain FVIII levels >50% for 1 wk).	Not stated	No bleeding complications, thrombotic events, thrombotic microangiopathies, or deaths
	13.5 y with severe hemophilia A	Posterior spinal fusion	**Preoperative:** 120 IU/kg rFVIII × 9. **Perioperative:** Multiple doses of FVIII (to maintain FVIII levels >50% for 1 wk).		
Vagrecha et al. 2020 [36]	Age and hemophilia A severity were not defined for the people with hemophilia A who underwent major surgery	Knee arthroplasty	**Perioperative:** rFVIII	Postoperative bleeding led to emicizumab discontinuation, despite FVIII replacement and absence of an FVIII inhibitor	No thrombotic events or deaths
Santagostino et al. 2019^b^ [22]	Four individuals ≥12 y of age with severe hemophilia A receiving emicizumab 1.5 mg/kg once weekly or 3 mg/kg once every 2 wk	Knee arthroscopic synovectomy	**Preoperative:** 55 IU/kg FVIII × 1 **Operative:** FVIII during the first 48 h **Postoperative:** FVIII for the first 7 days; FVIII infusions continued for 14–18 d. Antifibrinolytic use in 1 undefined individual.	Not stated	No bleeding or thrombotic complications
		Biceps femoris tear repair	**Operative:** 99 IU/kg FVIII during the first 48 h **Postoperative:** 225 IU/kg FVIII for the first 7 d; FVIII infusions continued for 14–18 d. Antifibrinolytic use in 1 undefined individual.		
		Total ankle arthroplasty	**Operative:** 187 IU/kg FVIII during the first 48 h **Postoperative:** 330 IU/kg FVIII for the first 7 d; FVIII infusions continued for 14-18 d. Antifibrinolytic use in 1 undefined individual.		
		Total hip replacement	**Operative:** 200 IU/kg FVIII during the first 48 h. **Postoperative:** 522 IU/kg FVIII for the first 7 d; antifibrinolytic use in 1 undefined individual.		

B/L, bilateral; CAD, coronary artery disease; EHL, extended half-life; F, factor; FVIII:C, factor VIII clotting activity; i.v., intravenous; NASH, nonalcoholic steatohepatitis; pd, plasma-derived; rFVIII, recombinant factor VIII; SHL, standard half-life; TXA, tranexamic acid; VWF, von Willebrand factor.

a Inhibitor status not defined for single individual undergoing major surgery. Due to the use of rFVIII, it is unlikely that they have inhibitors.

b Cohort included in the Kruse-Jarres 2022 publication.

The majority of publications reported on those with severe disease (13/19 [68.4%]) [[23], [24], [25],^27^,[29], [30], [31], [32], [33], [34],^37^,^39^,^41^], with 3 (15.8%) reporting on all severities of hemophilia A [26,36,38]. Three (15.8%) publications did not state the hemophilia A severity of all people with hemophilia A in the analysis [28,35,40]. Eight (42.1%) publications reported on people with hemophilia A who had comorbidities prior to surgery (*n* = 8), including, but not limited to, hepatitis C infection, human immunodeficiency virus infection, coronary artery disease, nonalcoholic steatohepatitis, and lymphoma [24,25,29,[31], [32], [33],^40^,^41^].

A total of 72 surgeries in people with hemophilia A without FVIII inhibitors were reported across 16 (84.2%) publications [[23], [24], [25], [26], [27], [28], [29],[31], [32], [33],[36], [37], [38], [39], [40], [41]], and the type of procedure was described for 36 surgeries in 14 (73.7%) of these publications [[24], [25], [26], [27], [28], [29],[31], [32], [33],^36^,^37^,[39], [40], [41]]. One of these publications described the type of surgery for 5 procedures, but did not state the type of major surgery for the remaining 13 procedures [39]. The number and types of major surgeries performed in people with hemophilia A without FVIII inhibitors were not clearly stated in the 3 (15.8%) remaining publications [30,34,35].

The weight of the person with hemophilia A at time of surgery was not reported in any of the publications.

### 3.2 Surgical experience in people with hemophilia A without FVIII inhibitors receiving emicizumab

Nine (47.4%) publications reported at least 1 orthopedic surgery [24,26,27,32,36,37,[39], [40], [41]], which included the following reported procedures (*n* = 22 [30.6%] orthopedic procedures; *n* = 51 [70.8%] procedures overall): arthroplasty (which includes joint replacement, fusion and endoprosthesis), and open reduction and internal fixation. Two additional publications also reported on orthopedic surgeries only, but the number of surgeries performed in people with hemophilia A without FVIII inhibitors was not stated [34,35]. Five (26.3%) publications reported on both orthopedic and other major surgeries (*n* = 27 [37.5%] procedures). Other major surgeries included, but were not limited to, splenectomy and screw fixation for a femoral neck fracture [26,27,37]. Five (26.3%) publications described other major surgical procedures only (*n* = 5 [6.9%] procedures), which included cleft palate correction [28] and cardiac surgery [25,29,31,33]. The type of major surgery performed was not reported at all in 2 publications (*n* = 16 [22.2%] procedures) [30,38]. Major surgeries were defined as per the classification in Santagostino et al. 2015 [42] in both of these publications. Two publications reported the type of procedure for some people with hemophilia A, but not all [39,40]. An additional publication did not report the type of major surgery, and the number of procedures performed in people with hemophilia A without FVIII inhibitors was not defined either [30]. The majority of procedures (33/72 [45.8%]) were performed at a specialized hemophilia treatment center [25,26,28,29,32,[36], [37], [38],^40^]. No publications reported any details on general anesthesia or the requirement for respiratory assistance.

### 3.3 Use of factor replacement

All procedures (*n* = 66 [91.7%]), apart from 6, were managed with FVIII replacement across the perioperative period (reported in 16 publications) [[23], [24], [25], [26], [27], [28], [29],[31], [32], [33],[36], [37], [38], [39], [40], [41]]. Where described, FVIII activity levels of between 50% and 120% for 3 to 20 days were aimed for during the perioperative period [25,27,31,33,37,40,41]. The specific chromogenic assay used to measure FVIII activity levels was not reported by the majority of publications; however, one reported the use of bovine chromogenic assay [29]. The additional 3 publications, in which the number of procedures in people with hemophilia A without FVIII inhibitors was not clear, also reported the use of FVIII replacement across this period [30,34,35]. Where described, preoperative FVIII replacement was given at a dose of 25 to 85 IU/kg [26,28,32,33,37,39,41], or as a single bolus of 4000 IU [24]. Operative management mostly involved the use of FVIII replacement, and this was reported in 8 (42.1%) publications (*n* = 18 [25.0%] procedures) [24,25,29,[31], [32], [33],^40^,^41^]. Dosage and administration frequency during surgery were not widely described, but where reported included a single bolus of 25 to 79 IU/kg (*n* = 16 procedures) [25,[31], [32], [33],^40^,^41^], a 3 to 4 IU/kg per hour infusion (*n* = 2 procedures [invasive aortic valve replacement and total arthroplasty of the elbow]) [24,29], or a single dose of 79 IU/kg (*n* = 1 procedure [an urgent bilateral osteosynthesis of the tibia and fibula]) [41]. One additional publication noted the operative use of FVIII replacement, if required, but the number of cases in which this was administered was not defined [39]. FVIII replacement was also administered at 15 to 55 IU/kg every 8 to 48 hours postoperatively for 1 to 29 days, where described [[24], [25], [26],^28^,^29^,^31^,^33^,^37^,[39], [40], [41]].

One publication reported the preoperative use of plasma-derived von Willebrand concentrate (73 μg/kg × 12 doses) for 1 intracranial ventricular shunt revision, which was also managed in conjunction with multiple doses of FVIII replacement [27].

One cardiopulmonary bypass grafting surgery was performed using emicizumab alone, with FVIII replacement available on standby [25]. However, recombinant FVIII replacement was eventually administered at the end of the procedure to maintain a postoperative FVIII level of 100%.

Two publications did not report the details of preoperative management for all of the procedures described (*n* = 18 [25.0%]) [38,40]. Two of the 8 procedures, for which preoperative management was not stated, were managed with postoperative factor replacement to treat bleeds [38].

### 3.4 Use of antifibrinolytics

Twenty-five (34.7%) procedures involved the use of antifibrinolytics. The use of tranexamic acid was not extensively described in the publications included in the review; however, 4 publications described its use in 15 (20.8%) procedures [28,33,37,41]. One publication, which reported a single aortic root abscess and aortic regurgitation, described operative usage of tranexamic acid. Here, tranexamic acid was administered at a dose of 2.07 g, followed by an infusion of 10 mg/kg per hour [33]. Tranexamic acid was also administered during a cleft palate correction, but no additional information regarding the dose or administration was reported [28]. An additional publication reported either oral or intravenous administration postoperatively, at a dose of 0.5 g for the one pediatric individual included, and 1 g for adult individuals, for a period of between 7 and 14 days [37]. Tranexamic acid (continuous infusion for an initial 8 hours) was also administered on the day of all 7 procedures reported in another publication [41].

An unspecified antifibrinolytic was used postoperatively following an orthopedic surgery, in a publication reporting on 4 procedures overall [22]. An additional publication noted that antifibrinolytics were used perioperatively for all people with hemophilia A undergoing surgery, with the exception of those undergoing urological procedures involving the uroepithelium (*n* = 9 [12.5%] procedures) [40].

Antifibrinolytics were not used in isolation and were utilized in conjunction with FVIII replacement in all procedures described above.

### 3.5 Use of anticoagulants and antiplatelets

Five (26.3%) publications reported the use of anticoagulants and/or antiplatelets during the operative period (*n* = 5 [6.9%] procedures). One individual was given an antiplatelet drug immediately after invasive aortic valve replacement [29]. In a publication reporting on 1 procedure (further described below), enoxaparin and edoxaban were used postoperatively for an individual who had undergone cardiac surgery and developed a left atrial appendage thrombus [31]. The same individual was treated with heparin boluses during surgery [31]. A cardiopulmonary bypass grafting surgery originally planned to be performed with emicizumab alone, with FVIII replacement on standby (as mentioned above), was managed operatively with FVIII replacement and the addition of heparin (400 U/kg) and protamine (275 mg). This individual was also prescribed 81 mg aspirin daily postoperatively [25]. Four operative heparin titrations (28,000 U, followed by 3 × 10,000 U) were also administered during the aortic root abscess and aortic regurgitation, which ultimately required further intervention [33]. In another publication reporting on 10 procedures, one individual was treated with a single dose of low molecular weight heparin following a laparoscopic single anastomosis gastric bypass. Due to a bleed of gastrointestinal origin, heparin treatment was stopped [40].

### 3.6 Additional postoperative management

Postoperative physical therapy was not reported by the majority of the publications included in the analysis (15/19 [78.9%]). Physical therapy was described in a small number of cases [26,30,34,37,39,41]. Castaman et al. [37] reported that rehabilitation was carried out under emicizumab prophylaxis only after a total knee replacement with forced extension (1 of the 6 procedures described), and no bleeds or additional complications occurred. Two individuals who had undergone surgery in Carulli et al. [34] were sent to the rehabilitation ward at the same hospital shortly after major surgery. The specific procedures for people with hemophilia A without FVIII inhibitors were not described.

Ten (52.6%) publications reported hospitalization postsurgery (*n* = 22 [30.6%] procedures); the duration of stay ranged from 2 to 58 days [25,26,28,29,31,32,34,37,40,41]. Shorter hospital stays were associated with, but not limited to, a turbinate reduction (2 days) [37], cleft palate correction (2 days) [28], and right medial patellofemoral ligament reconstruction (3 days) [26]. The remaining publications did not report on hospitalization. One publication noted that hospitalization time was as expected for all 6 procedures described [37], and another indicated that hospitalization was as planned for the single procedure reported [28]. The remaining publications did not state whether duration of hospitalization was as expected for the procedure.

### 3.7 Bleeding events and blood transfusions

Seventeen (89.5%) publications reported on the occurrence or absence of bleeding events; of these, 9 (47.4%) publications stated that no bleeding complications occurred during or after surgery in any individual without FVIII inhibitors (*n* = 22 [30.6%] reported procedures) [[23], [24], [25],^27^,^28^,^30^,^32^,^34^,^37^]. Following 56 (77.8%) procedures (reported across 14 publications), no postoperative bleeding events occurred. The remaining publications (2/19 [10.5%]) did not report on any bleeding events associated with surgery in people with hemophilia A without FVIII inhibitors [29,35].

Although the absence or occurrence of bleeding events were reported by the majority of the publications (89.5%), the bleeding events were not widely described in detail, particularly with regard to blood loss; however, bleeding events were described by 8 (42.1%) publications (*n* = 15 [20.8%] procedures; Table 3). Of the people with hemophilia A who experienced bleeding events, none of the reported outcomes prior to surgery could definitively be used to predict the occurrence of a bleeding event. For instance, both orthopedic and nonorthopedic surgery resulted in bleeding events, and both people with and without reported comorbidities experienced bleeds.

**Table 3:** Reported bleeding events in people with hemophilia A without factor VIII inhibitors receiving emicizumab undergoing major surgery.

Publication	Surgery type	Bleeding event
Abraham et al. 2023 [39]	Right total knee replacement	One breakthrough bleed, treated with rFVIII
	B/L total knee replacement	Two breakthrough bleeds, treated with rFVIII
	Left total knee replacement	One breakthrough bleed, treated with rFVIII
	Right thigh pseudotumor above-knee amputation	One stump bleed, treated with rFVIII
	B/L total knee replacement	Three breakthrough bleeds, treated with rFVIII
Buckner et al. 2023 [38]	Total knee replacement	Bleeding event treated with SHL FVIII
	Reconstruction of the anterior cruciate ligament of the knee	Bleeding event treated with SHL FVIII
Cohen et al. 2023 [40]	Laparoscopic single anastomosis gastric bypass	Major bleeding of gastrointestinal origin
	Retrograde intrarenal surgery with stent insertion	Clinically relevant non-major bleeding
	Total hip replacement	Major bleeding that required daily FVIII treatment at home for a period of 11 days
Rener et al. 2023 [41]	Urgent bilateral osteosynthesis of the tibia and fibula	One major bleed, which resulted in a blood transfusion
Sreedhar et al. 2023 [31]	Cardiac surgery	High blood outlet in the drains 6 h postsurgery. Continuous infusion of FVIII was used to maintain FVIII:C >100 IU/dL
Cusano et al. 2022 [33]	Aortic root abscess and aortic regurgitation	∼1600 mL of blood loss via chest tubes 2 h postsurgery. Multiple doses (2 g each) of fibrinogen concentrate and multiple doses of platelets were administered before surgical intervention was required
Lewandowska et al. 2021 [26]	Arthroscopic synovectomy of the elbow with nerve transposition	Postoperative bleeding (believed to have been aggravated by physical therapy) into the soft tissue/muscles of the left forearm on postoperative day 8
Vagrecha et al. 2020 [36]	Knee arthroplasty	Recurrent bleeding into the replaced joint. Emicizumab was discontinued

B/L, bilateral; F, factor; FVIII:C, factor VIII clotting activity; rFVIII, recombinant factor VIII; SHL, standard half-life.

Five (26.3%) publications specifically reported on blood transfusions [25,33,37,39,41], and of these, 3 reported that blood transfusions were required. The remaining publications (14/19 [73.7%]) did not report whether blood transfusions were required.

One publication noted that bleeding was as predicted for all 6 procedures described [37], but none of the remaining publications commented on whether bleeding was as expected. None of the publications commented on whether the likelihood and extent of bleeding was commensurate with that expected in the general population.

### 3.8 Thrombotic events and deaths

Nine (47.4%) of the publications reported that no thrombotic or thromboembolic events, and no deaths, occurred [23,[26], [27], [28],[35], [36], [37],^39^,^40^]. Seven (36.8%) of the included publications did not report on these outcomes [24,25,29,30,33,34,38]. Two publications reported that no thrombotic events occurred for some individuals undergoing surgery, but did not report these outcomes for the remaining people with hemophilia A in the cohort [32,41].

One thrombotic event was reported: a left atrial appendage thrombus was diagnosed 14 days postsurgery in a publication reporting on one cardiac surgery, as described previously, which was treated with enoxaparin followed by edoxaban with a bolus of standard half-life FVIII replacement. No further interventions were required [31].

One publication reported the use of nonpharmacologic methods to prevent thrombosis, but did not state in which people with hemophilia A these were used [41]. None of the remaining publications reported on the use of mechanical intervention to prevent the occurrence of thrombotic events.

## 4 Discussion

To our knowledge, this is the first literature review to describe the broad, global experiences of people with hemophilia A without FVIII inhibitors treated with emicizumab who have undergone major surgery in 1 consolidated report.

While there were only 20 publications included in the review, and inconsistencies and gaps in the reporting of information, this analysis in people with hemophilia A without FVIII inhibitors undergoing surgery is consistent with the previously reported safety profile and efficacy/effectiveness of emicizumab prophylaxis [[9], [10], [11], [12], [13],[15], [16], [17], [18], [19], [20],^43^]. Most major surgeries were managed with factor replacement during the perioperative period. There were no AEs reported that were explicitly deemed related to the use of factor replacement in conjunction with emicizumab in a surgical context, although this was possibly not discussed by the relevant publications.

Hemostasis was effectively controlled for most surgeries using factor replacement throughout the entire perioperative period, which aligns with the recommendations outlined by the WFH [2] and the National Hemophilia Foundation’s Medical and Scientific Advisory Council (MASAC) [44] for people with hemophilia A who are currently on emicizumab prophylaxis. Based on the low number of bleeding events noted in this review, it could be suggested that in order to attain optimal surgical outcomes, following these recommendations may be crucial.

Our search found that only a small number of publications reported the use of antifibrinolytics. Tranexamic acid is most often recommended and administered during minor dental procedures [2,[45], [46], [47]], so these low numbers would be expected in a population undergoing major surgery.

Bleeding events were reported in 15 people with hemophilia A undergoing major surgery while treated with emicizumab in 8 (42.1%) publications (*n* = 15 [20.8%] procedures); however, it should be acknowledged that bleeding to some extent would be expected in the general population during and after major surgery [[48], [49], [50]], although this may not be to the same degree as people with hemophilia A would experience.

Although 2 individuals reported discontinuation of emicizumab therapy, there were no additional instances where this was reported, suggesting that the majority of people with hemophilia A remained on emicizumab prophylaxis following surgery.

One thrombotic event was reported, which corresponds with the safety outcomes previously reported for people with hemophilia A without FVIII inhibitors treated with emicizumab [15,[51], [52], [53]]. However, the publication in which this event was reported does not explicitly state whether it was related to the use of emicizumab in conjunction with factor replacement [31], so this possibility cannot be ruled out. We found that 8 of the publications did not report on the occurrence or absence of thrombotic events; it would be expected that the incidence of a serious event such as this would always be reported.

Notably, we found that there are very few publications that report on physical therapy following surgery in emicizumab-treated people with hemophilia A without FVIII inhibitors, limiting the extent to which we could report on this outcome. The importance of physical therapy and exercise for people with hemophilia A is widely recommended. The MASAC guidelines indicate that physical therapy should be an important part of the rehabilitation process following surgery, and they specify recommendations for those who have undergone synovectomy and total knee replacement [54], which were surgeries reported in our analysis. These recommendations are also aligned with the WFH guidelines, where gradual rehabilitation with a physical therapist is recommended, particularly following orthopedic surgery [2]. Although we found that 2 bleeding events were reported to be related to physical or occupational therapy following surgery, the guidelines above indicate that the benefits of this type of management may outweigh the risks [2,54]. For these reasons, it could be considered that in the publications that did not report on physical therapy, it may have taken place as standard of care. Notably, this seems to be a gap in the literature and could be a future direction for research, such as exploring how current therapies support postoperative rehabilitation and recovery of people with hemophilia A after major surgery. Information on whether physical therapy could continue successfully postoperatively without disruption may also be helpful.

Little information on hospitalization following surgery was provided, indicating that more detailed reports on the postsurgical period are required for this population. Notably, length of hospitalization may be dependent on the country and/or healthcare system in which the procedure takes place. Planned hospitalization time, to some degree, would also be expected following major surgery in the general population [[55], [56], [57]].

### 4.1 Limitations

We found that many of the publications identified did not report on a number of outcomes of interest, exemplifying the current lack of reported information surrounding, for example, the complementary use of anticoagulants and antifibrinolytics during the perioperative period, in addition to the use of postoperative physical therapy, for people with hemophilia A without FVIII inhibitors undergoing major surgery. It is unclear whether this information was simply unreported, or whether it did not take place. This also made it difficult to assess the outcomes that may have been predictors of bleeding events. The lack of consistently reported data also necessitated a narrative literature review as no meta-analysis could be performed.

The limitations of our review also include the high number of case studies evaluated, which may overestimate the risk of AEs and be less likely to represent the population as a whole: cases that are deemed uneventful may be less likely to have been reported or published. Additionally, in some publications, it is not clear whether bleeding or AEs are not reported at all, or whether they are not reported because they did not occur.

Our analysis did not include publications for which it was unclear whether some procedures were performed in people with hemophilia A with or without FVIII inhibitors, meaning that some information on the surgical experience of people with hemophilia A without FVIII inhibitors may not have been included. Furthermore, the formation of FVIII inhibitors following surgery was not explored in this analysis. As data were mostly extracted from publications authored by physicians at specialized hemophilia treatment centers, publication bias may also limit our analysis. Surgeons at specialized centers are likely to be more experienced in performing surgery in people with hemophilia A, and better at preoperative planning and coordinating with a hematologist. However, this may not be standard practice in all treatment centers and/or locations. The inconsistencies and heterogeneity in the reporting of the different outcomes, due to the range of people with hemophilia A, settings, operators, and variability in practice patterns may have also limited the overall analysis.

It is likely that the summary of procedures discussed here are generally representative of the typical procedures that people with hemophilia A undergo, and the outcomes are consistent with what has been observed in clinical trials [22,23]. While there is currently a lack of consistency in the reporting of specific outcomes of interest, particularly with regard to AESIs, our analysis indicates that the reported experience of major surgery in people with hemophilia A without FVIII inhibitors on emicizumab is increasing. However, more data are required in order to generate well-established guidelines for the management of this population during major surgery. In addition to this, the growing experience regarding the management and support of people with hemophilia A on emicizumab will allow further analysis of these outcomes, helping to support physicians and the hemophilia community when making treatment decisions, particularly surrounding the management of major surgical procedures.

## 5 Conclusions

The previously reported safety profile and efficacy/effectiveness of emicizumab prophylaxis in people with hemophilia A without FVIII inhibitors are supported by the findings of this literature review. However, this review provides evidence that there is a need for greater consistency among the reporting of surgeries for people with hemophilia A. Many of the preselected outcomes were not reported by the publications included in the review or were reported inconsistently. Factor replacement was used to manage most major surgeries described in people with hemophilia A without FVIII inhibitors, and the safety and tolerability of emicizumab does not differ from that reported outside of a surgical setting. Postoperative bleeding was reported in 15 out of the 72 procedures described. Although physical therapy is recommended by the guidelines, this was not widely reported on, and further information is warranted. This review provides a consolidated collection of the reported experiences of emicizumab-treated people with hemophilia A without FVIII inhibitors undergoing major surgery, and highlights that increased granularity of data collection and greater consistency in data reporting, arguably for any prophylaxis, on concomitant treatment and postoperative care are needed. We also hope that this review will help to better inform the development of any guidance related to perioperative management of people with hemophilia on any current or future prophylactic agent.
