# Supplementary material for: A literature review of major surgery experience with emicizumab in people with hemophilia A without factor VIII inhibitors
Source: Res Pract Thromb Haemost. 2025 Jan 31;9(1):102693. doi: 10.1016/j.rpth.2025.102693 (PMC11909758; doi:10.1016/j.rpth.2025.102693)
Supplement: Supplementary Material [file mmc1.docx]

**Supplementary Material**

**Supplementary Table 1.** Search methodology

| **Search**  **number** | **Query** | **Results** |
| --- | --- | --- |
| 1 | "emicizumab"[Supplementary Concept] OR Hemlibra[all] OR "ACE910" [all] OR "ACE-910"[all] OR "ACE 910"[all] OR "emicizumab-kxwh"[all] OR "emicizumab" [all] | 639 |
| 2 | ("Hemophilia A"[Mesh] OR "Hemophilia A"[tiab] OR "Hemophilia"[tiab] OR "Haemophilia"[tiab] OR "Congenital Hemophilia A"[tiab] OR "Classic Hemophilia"[tiab] OR "Autosomal Hemophilia A"[tiab] OR "Factor VIII Deficiency"[tiab] OR "Factor 8 Deficiency"[tiab] OR "Congenital Factor VIII Deficiency"[tiab] OR "Haemophilia A"[tiab] OR "Congenital Haemophilia A"[tiab] OR "Classic Haemophilia"[tiab] OR "Autosomal Haemophilia A"[tiab]) | 29,976 |
| 3 | ("invasive"[tiab] OR "procedure*"[tiab] OR "surg*"[tiab] OR "Operat*"[tiab] OR "surgery"[Subheading] OR "General Surgery"[MeSH] OR "Surgical Procedures, Operative"[MeSH] OR "Arthroplasty"[MeSH] OR "Arthroplast*"[tiab] OR "synovectomy"[MeSH] OR "synovectom*"[tiab] OR "arthrodesis"[MeSH] OR "arthrodes*"[tiab] OR "cardiopulmonary bypass surgery"[tiab] OR "amputation, surgical"[MeSH] OR "amputat*"[tiab]) | 6,876,620 |
| 4 | #1 AND #2 AND #3 | 107 |
| 5 | "Postoperative management"[tiab] OR "Physical Therapy Modalities"[MeSH] OR "physical therap*"[tiab] OR "physiotherapy"[tiab] OR "joint surgery"[tiab] OR "Blood Coagulation Factor Inhibitors"[MeSH] OR "antagonists and inhibitors"[Subheading] OR "inhibit*"[tiab] OR "Factor VIII/antagonists and inhibitors"[MeSH] OR "Anticoagulants"[MeSH] OR "Anticoagulant*"[tiab] OR "Anti coagulant*"[tiab] OR "Anti-coagulant*"[tiab] OR "Heparin"[MeSH] OR "Heparin"[tiab] OR "Venous Thrombosis"[Mesh] OR "Deep vein thrombosis"[tiab] OR "Deep venous thrombosis"[tiab] OR "DVT"[tiab] OR "FVIII"[tiab] OR "F VIII"[tiab] OR "Factor VIII"[tiab] OR "Factor 8"[tiab] OR "Factor Eight"[tiab] OR "Factor VIII"[MeSH] | 3,504,299 |
| 6 | #4 AND #5 | 103 |
